# RNF4-mediated SUMOylation is essential for NDRG2 suppression of lung adenocarcinoma

**DOI:** 10.18632/oncotarget.8663

**Published:** 2016-04-09

**Authors:** Jicheng Tantai, Xufeng Pan, Dingzhong Hu

**Affiliations:** ^1^ Department of Thoracic Surgery, Shanghai Chest Hospital, Shanghai Jiao Tong University, Shanghai, China

**Keywords:** NDRG2, RNF4, SUMOylation, tumorigenesis, STUbL

## Abstract

N-Myc downstream-regulated gene 2 (NDRG2) protein is a tumor suppressor that inhibits cancer growth, metastasis and invasion. The ubiquitin ligase RNF4 integrates signaling by SUMO and ubiquitin through its selective recognition and ubiquitination of SUMO-modified proteins. We evaluated NDRG2 SUMOylation in lung adenocarcinoma cells and its underlying molecular mechanism. The results showed that NDRG2 is covalently modified by SUMO1 at K333, which suppressed anchorage independent adenocarcinoma cell proliferation and tumor growth. In human lung adenocarcinomas cells, RNF4 targeted NDRG2 to proteasomal degradation by stimulating its SUMOylation. Endogenous RNF4 expression was increased in human lung adenocarcinomas cells, and there was a concomitant upregulation of SUMO. These findings indicate that SUMOylation of NDRG2 is necessary for its tumor suppressor function in lung adenocarcinoma and that RNF4 increases the efficiency of this process.

## INTRODUCTION

Lung cancer was the most commonly diagnosed cancer and the leading cause of cancer death among males worldwide in 2008. Among females, it was the fourth most commonly diagnosed cancer and the second leading cause of cancer death [1]. The best approach for the treatment of lung cancer is currently complete surgical removal of the tumor and adjacent lymph nodes. However, the efficacy of this therapeutic approach alongside hormone therapy, radiotherapy and chemotherapy is limited [2]. Consequently, there is an urgent need to explore new targets and therapeutic approaches to the treatment of lung cancer.

Tumor suppressors are integral to preventing the accumulation of harmful and potentially oncogenic mutations. N-myc downstream-regulated gene 2 (NDRG2) is a 41-kDa cytoplasmic protein of NDRG family. It is involved in multiple biological processes, including cell growth, differentiation, and apoptosis [3]. Evidence suggests that NDRG2 may act as a tumor suppressor in various cancers. For example, NDRG2 expression is deficient in melanoma, glioblastoma, thyroid cancer, colon cancer, and pancreatic cancer [4–8]. In addition, NDRG2 reportedly inhibits proliferation of certain tumor cells [9, 10]. The actions of NDRG2 in lung cancer remain unclear, however.

SUMOylation regulates cellular processes that include nucleocytoplasmic transport, gene transcription and DNA repair [11–14]. Desumoylating enzymes allow dynamic regulation and have become an important target for therapeutics [15]. Recently, NDRG2 was shown to be ubiquitinated by TRIM32 *in vitro*, and to accumulate in skeletal muscle and myoblasts in the absence of TRIM32. Overexpression of NDRG2 in myoblasts led to reduced cell proliferation and delayed cell cycle withdrawal during differentiation [16]. In this report, we present evidence that NDRG2 is also modified by SUMOylation catalyzed by RING finger protein 4 (RNF4) and that SUMOylation is required for NDRG2 regulated tumorigenesis in lung carcinoma cells.

## RESULTS

### NDRG2 SUMOylation suppresses anchorage-independent growth of lung adenocarcinoma cells

To conduct functional analyses, we used a lentiviral vector to generate A549 cell transfectants stably expressing wild-type (WT) NDRG2 or a K33R or K245R NDRG2 mutant. The expression of NDRG2 was comparable in all clones when assessed by western blotting, indicating that the two mutations did not affect the NDRG2 transcription rate and protein stability (data not shown).

**Figure 1:**
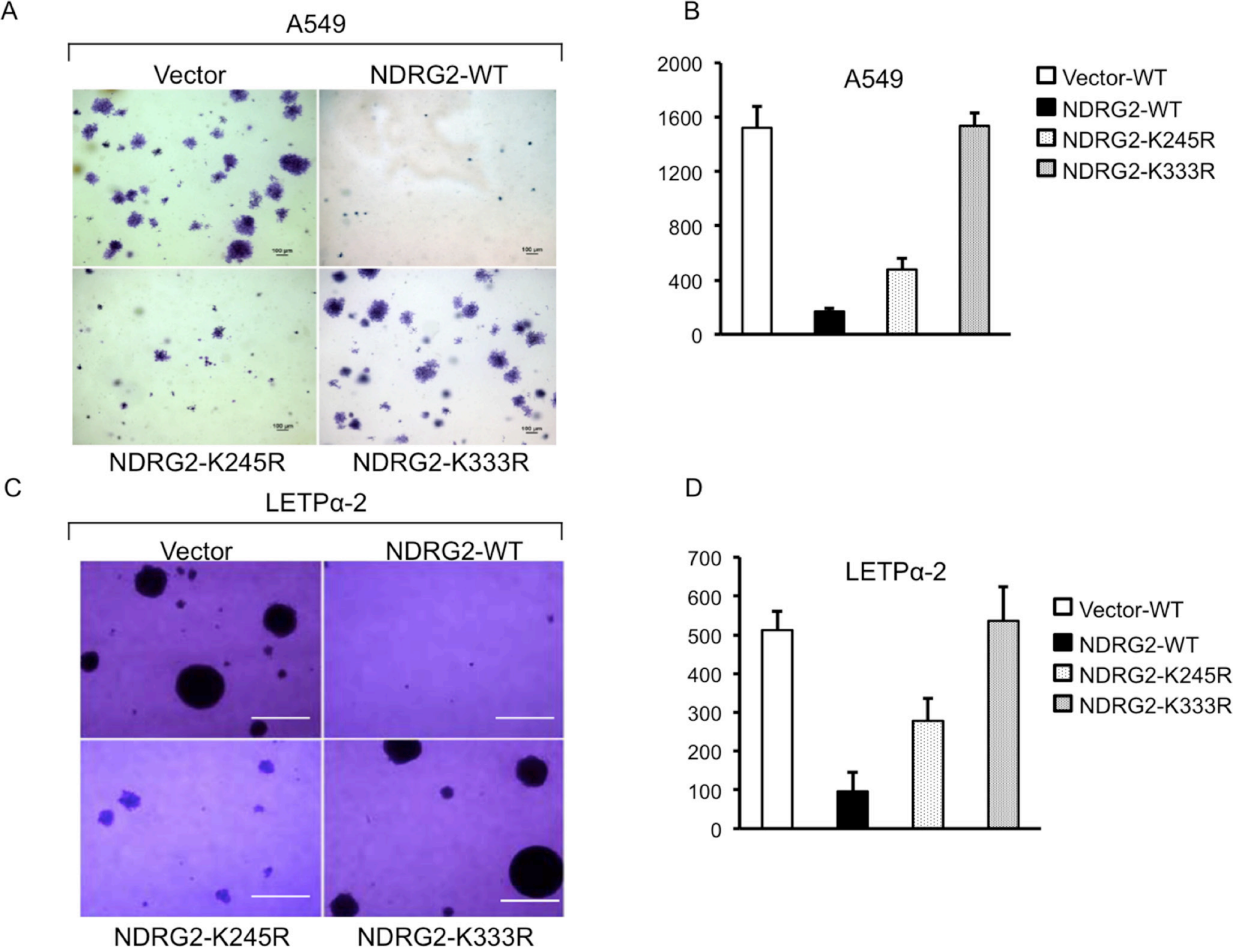
NDRG2 SUMOylation is essential for suppression of anchorage-independent growth of lung adenocarcinoma cells (**A–D**) Effects of wild-type NDRG2 (WT) and the indicated mutants on anchorage-independent growth of A549 (A, B) and LETPα-2 (C, D) cells assessed using soft agar colony-forming assays. Left panels: representative photomicrographs of the colonies. All images were identically processed and used the same scale bar (100 μm). Right panels: group data. Bars depict means ± SE. *P < 0.05.

To explore the effect of NDRG2 SUMOylation on the transforming potential of A549 cells, we performed soft agar colony-forming assays in the presence of 10% FBS to assess the cells' capacity for anchorage-independent growth. As expected, expression of NDRG2-WT inhibited colony formation by the cells. By contrast, NDRG2-K245R expression had no effect on anchorage-independent growth. The NDRG2-K245R transfectants produced colonies with cell numbers equivalent to those produced by the Vector-transfected cells (Figure 1A and 1B). Expression of NDRG2-K333R also suppressed growth, but to a lesser extent than NDRG2-WT. Similar results were obtained with LETP α-2 cells (Figure 1C and 1D).

### RNF4 reduces NDRG2 levels in lung adenocarcinoma cells

**Figure 2:**
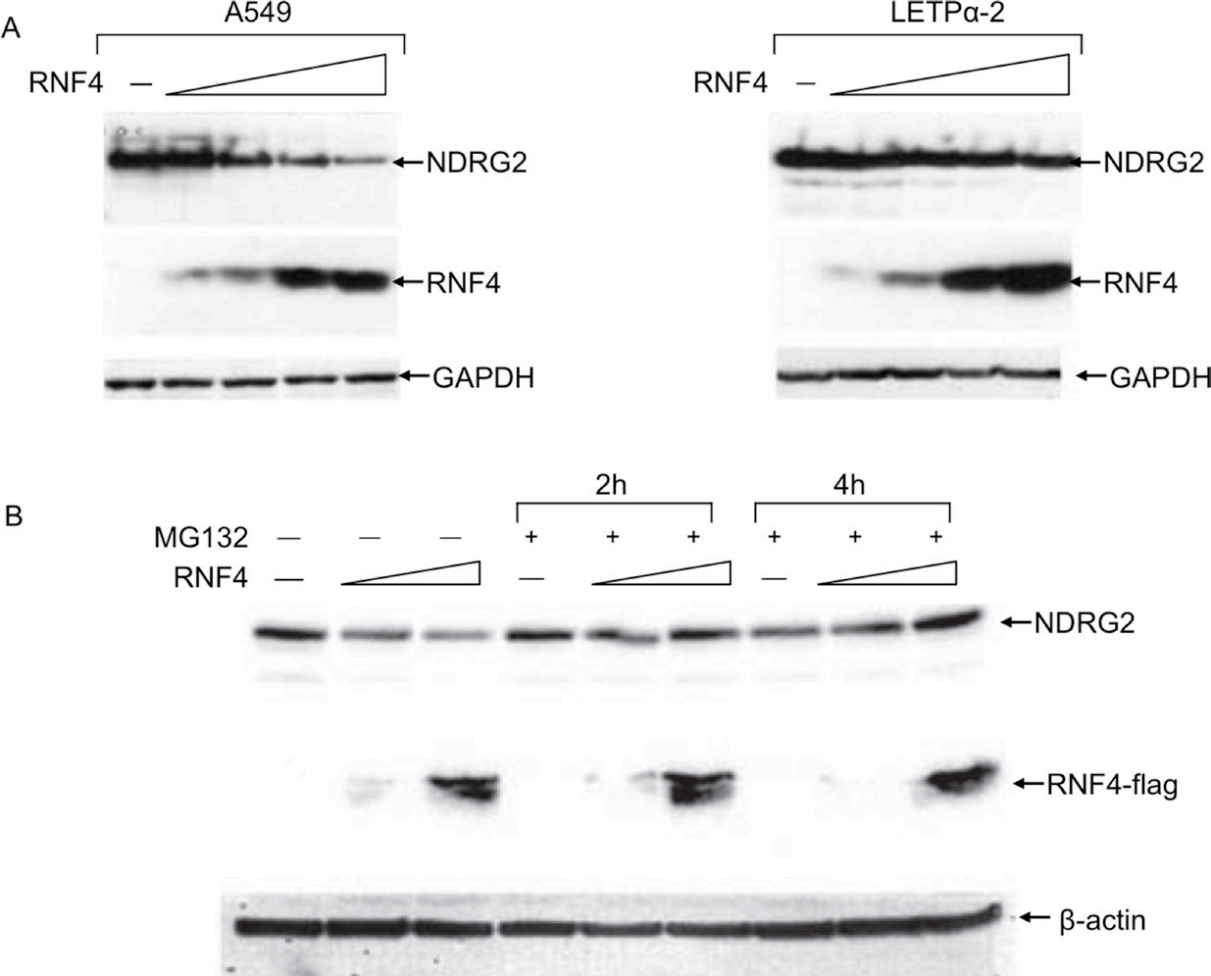
RNF4 expression induces NDRG2 proteasome-mediated degradation (**A**) WeA549 cells were transfected with increasing amounts of p14RNF4 expression plasmid. Protein extracts were fractionated using SDS-PAGE, and NDRG2 and RNF4 levels were analyzed by western blotting with anti-NDRG2 and anti-RNF4 antibodies. GAPDH was used as loading control. (**B**) A549 cells transfected with two levels of 3xFlag RNF4 expression plasmid were incubated with the proteasome inhibitor MG132 for 2 h or 4 h before lysis. Cellular extracts were immunoblotted with anti-NDRG2 and anti-Flag antibodies.

Increasing exogenous expression of RNF4 in these cells elicited a dose-dependent reduction in NDRG2 levels (Figure 2A). This effect of RNF4 was blocked by MG132 (Figure 2B), indicating that RNF4 reduced NDRG2 levels through degradation via proteasome. We therefore examined the ability of RNF4 to promote degradation of NDRG2 E268X and K245R SUMOylation mutants. The NDRG2-E268X bears a nonsense mutation disrupting the SUMO recognition sequence, while in NDRG2-K245R the SUMO acceptor lysine was replaced with an arginine. Neither RNF4 nor SUMO induced NDRG2-E268X or -K245R SUMOylation and degradation. This indicates that RNF4-mediated NDRG2 degradation requires intact SUMO consensus and acceptor sites (Figure 2C).

### RNF4 promotes NDRG2 SUMO-conjugation in lung adenocarcinoma cells

**Figure 3:**
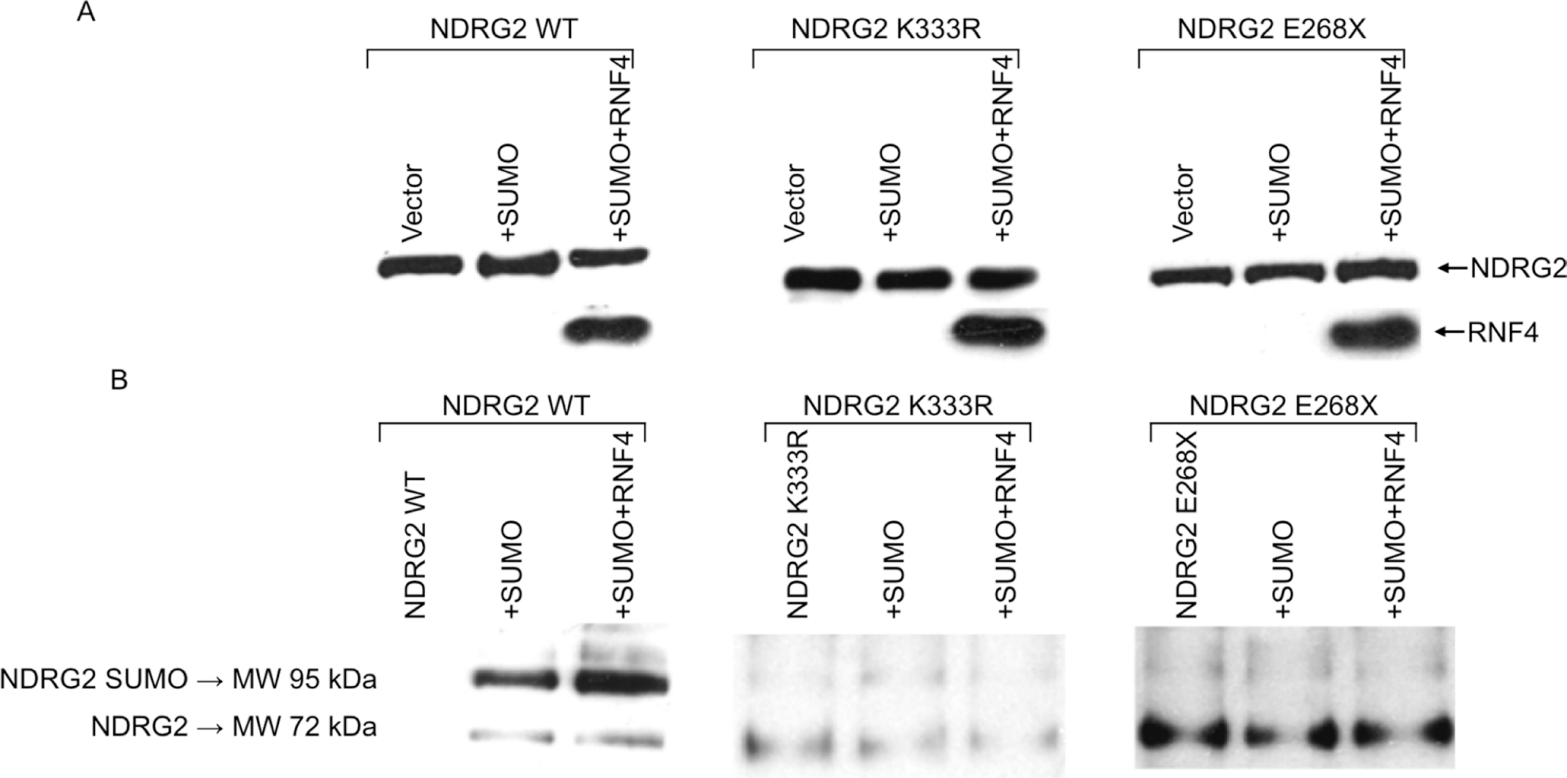
RNF4 expression increases SUMO-1 conjugation to NDRG2 in Nickel pull-down assays A549 cells transiently transfected with NDRG2-WT, K333R or K245R, alone or together with His6-SUMO1 or His6-SUMO1 plus RNF4, were treated with MG132. (**A**) Levels of exogenous NDRG2 and RNF4 expression were assayed by immunoblotting with anti-NDRG2 and anti-RNF4 antibodies. (**B**) Western blot analysis using anti-NDRG2 antibody of isolated His-tagged proteins collected using nickel nitrilotriacetic acid (Ni-NTA) agarose. Molecular weight markers and the positions of NDRG2 and His6-SUMO1-conjugated NDRG2 are shown on the left.

We next performed Nickel pull-down assays. A549 cells were transiently transfected with WT or mutant NDRG2, alone or with His-tagged SUMO1 and RNF4, in presence of MG132 (Figure 3A). Cell extracts were incubated with Ni-NTA agarose beads to isolate His-tagged SUMO-conjugated proteins. After elution, the proteins were analyzed by immunoblotting with anti-NDRG2 antibodies. As shown in Figure 3B, higher molecular weight species of NDRG2 were present when WT NDRG2 was expressed with SUMO1, and those species were selectively enriched by co-expression of RNF4 with SUMO. This indicates that RNF4 stimulated NDRG2 SUMOylation. Under the same experimental conditions, the NDRG2-K33R and NDRG2-E268X mutants were not SUMOylated, confirming that RNF4-induced SUMOylation of NDRG2 occurs at lysine 333 (Figure 3B).

**Figure 4:**
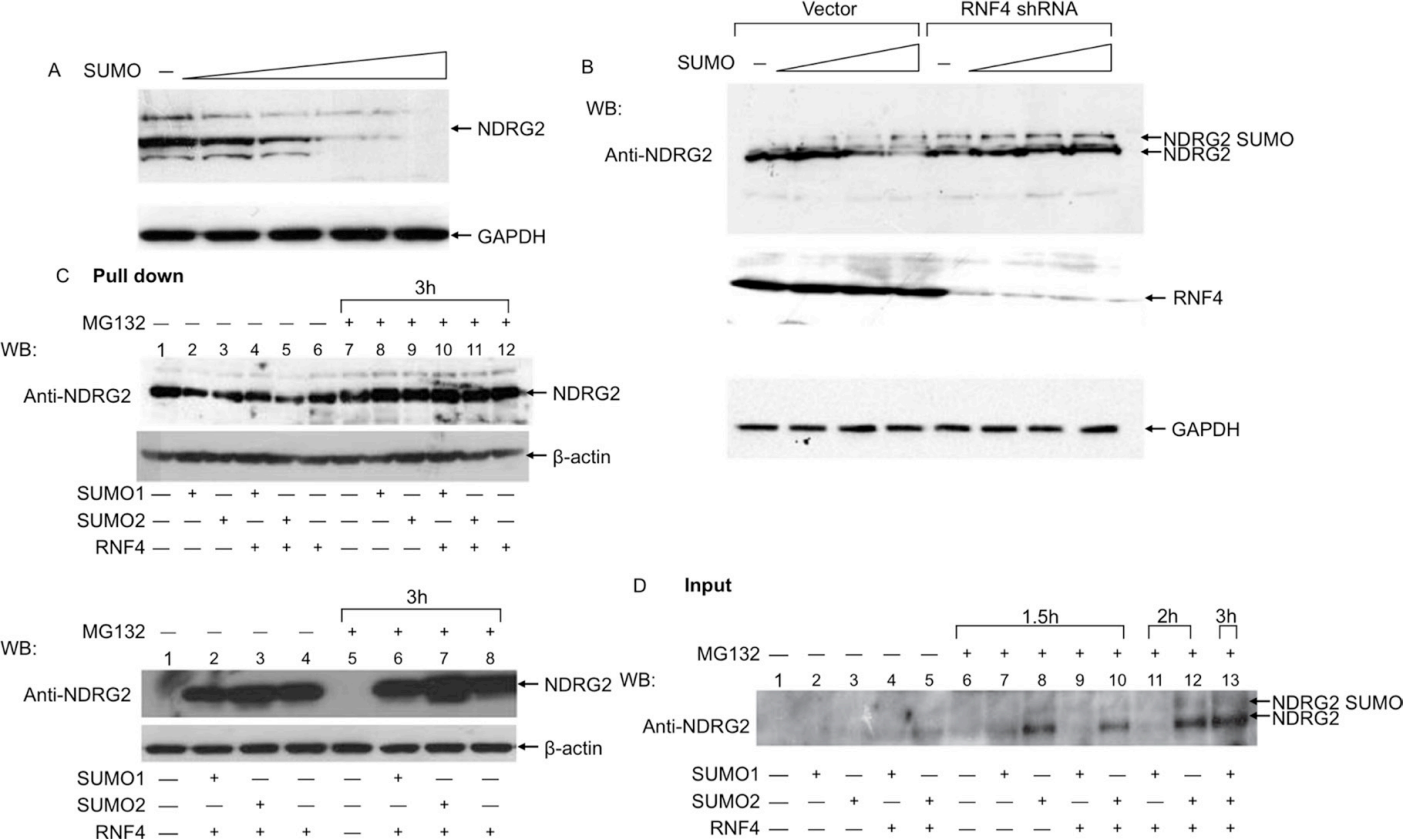
RNF4 and SUMO-1 expression induce endogenous NDRG2 SUMOylation and degradation in lung adenocarcinoma cells (**A**) A549 cells were transfected with a fixed amount of NDRG2 plasmid and increasing amounts of SUMO-1. NDRG2 levels were analyzed by immunoblotting with anti-NDRG2. (**B**) A549 cells were simultaneously transfected with a fix amount of NDRG2 plasmid and increasing amounts of SUMO1 alone or together with RNF4 siRNA or scrambled siRNA (negative control). Extracts collected in protein loading buffer were analyzed using anti-NDRG2 and anti-RNF4. Arrows indicate NDRG2 and SUMOylated NDRG2 species. GAPDH was used as loading control. (**C** and **D**). Nickel pull-down assays. A549 cells were transfected with plasmids encoding 3×Flag-RNF4, His6-SUMO-1 and His6-SUMO-2 alone or in combination, as indicated, after which they were treated with MG132 for 1.5, 2 or 3 h. Shown in **C** are western blots of His-tagged proteins pulled down using anti-NDRG2. Shown in D are western blots with anti-NDRG2 and anti-Flag showing expression of the transfected proteins.

We also evaluated the contribution of RNF4 to SUMO-mediated degradation of NDRG2 by depleting endogenous RNF4 in A549 cells, which abundantly express RNF4. We initially verified that in A549 cells NDRG2 was efficiently degraded by SUMO1 (Figure 4A). We then co-transfected A549 cells with a fixed amount of NDRG2 plasmid and increasing amounts of SUMO1 in presence of RNF4-targeting or scrambled siRNA. As shown in Figure 4B, NDRG2 degradation was inhibited by RNF4 knockdown, even though we could still observe the band corresponding to SUMO-conjugated NDRG2 (Figure 4B). It thus appears that RNF4 is necessary for efficient degradation of NDRG2.

Finally, we performed Nickel pull-down assays with A549 cells to evaluate NDRG2 SUMOylation status after co-transfection of SUMO1 or -2 plus RNF4. His-SUMO1- or His-SUMO2-linked NDRG2 was clearly detected in cells transfected with SUMO1/2 only when cells were treated with MG132 (Figure 4C and 4D). In addition, endogenous NDRG2 SUMOylation was more evident when RNF4 was co-expressed with SUMO2 and under conditions in which proteasome activity was inhibited (compare lane 8 with lanes 10, 12 and 13).

## DISCUSSION

SUMOylation is a post-translational modification involved in various cellular processes. Hundreds of SUMOylation substrates have been identified; most are nuclear and perinuclear proteins [17, 18], though some cytoplasmic proteins also reportedly exist in a SUMOylated form [19, 20]. NDRG2 is one of four members of the NDRG family [21]. It is downregulated in a variety of human tumors, which correlates with a improved prognosis in patients [22, 23]. This suggests NDRG2 may act to suppress carcinogenesis. In the present study, we confirmed that NDRG2 can be modified by SUMO1 in lung adenocarcinoma cells and that NDRG2 SUMOylation increases the protein's ability to inhibit lung cancer tumorigenesis.

**Figure 5:**
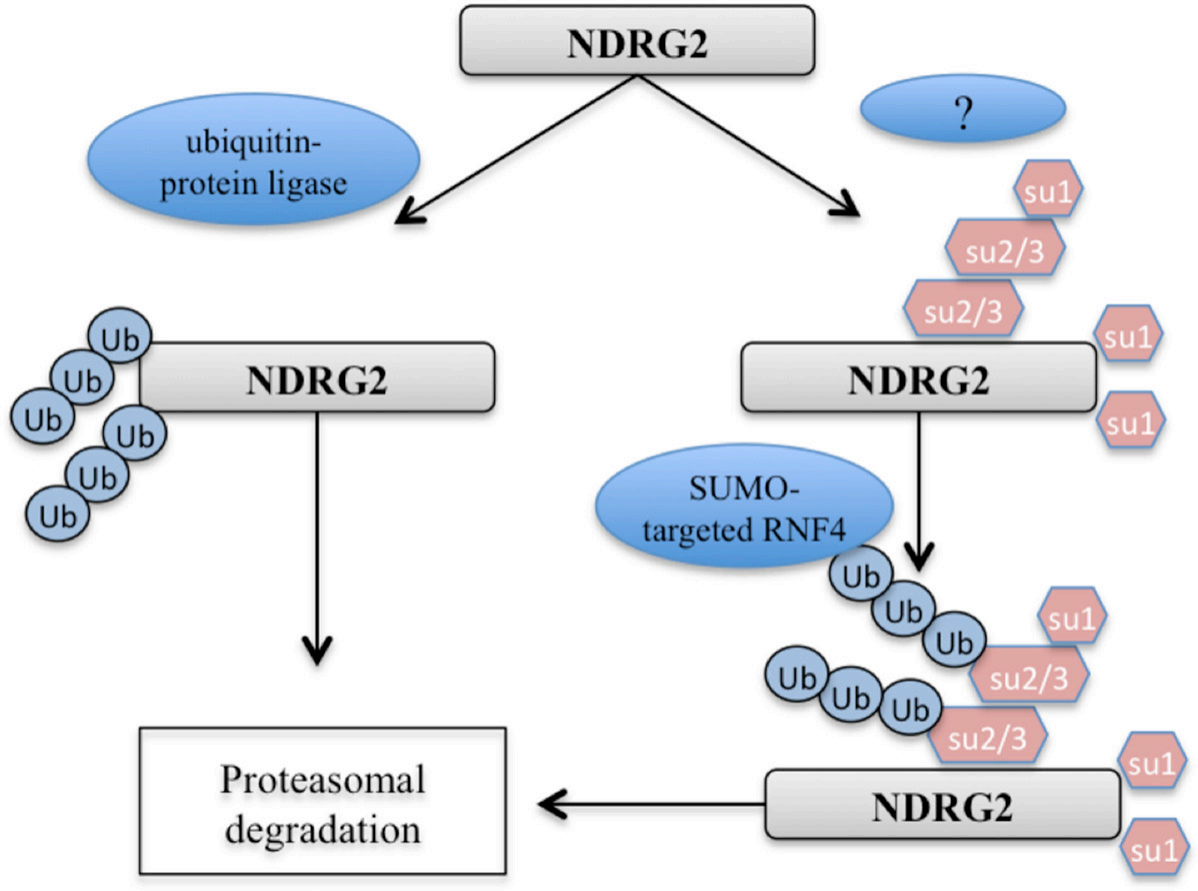
Model for the regulation of NDRG2 stability through SUMOylation

Protein modification by ubiquitin and Ubl (ubiquitin-like) proteins contributes to the regulation of cellular processes that include cell cycle progression, transcription, DNA repair and stress responses, among others, and their dysregulation has been implicated in cancer and aging [24–27]. Both ubiquitin and SUMO (small ubiquitin-related modifier) can form substrate-attached chains, in which one unit of the respective modifier is attached to a lysine residue of another. RNF4 is a SUMO-targeted E3 ubiquitin ligase with a pivotal function in the DNA damage response (DDR) [28]. RNF4 is also required for homologous recombination, indicating that balanced ubiquitination and de-ubiquitination of SUMOylated proteins is required for efficient DNA repair via homologous recombination [29, 30]. Recent studies also showed that RNF4 is recruited to sites of DNA repair, where it stimulates ubiquitylation and, at least in some cases, the turnover of repair factors [28–31]. Here, we showed that NDRG2 is preferentially SUMOylated by SUMO and that RNF4 increased the efficiency of this process. Our data illustrate a new approach to correlating the expression patterns of NDRG2, RNF4 and SUMO machinery in lung cancer (Figure 5). There is also potential cross-regulation between ubiquitin and SUMO [32]. As shown in Figure 3B, the NDRG2-K333R mutant cannot be modified with SUMO-1. Since ubiquitin conjugation of the K333R mutant is not possible, we can conclude that when conjugating NDRG2, SUMO-1 and ubiquitin do not compete for the same lysine residue.

In summary, our data reveal an unexpected regulatory mechanism in which SUMOylation increases the ability of NDRG2 to inhibit lung cancer tumorigenesis, and RNF4 targets NDRG2 to proteasomal degradation by stimulating its SUMOylation. Our findings may have important implications regarding cancer etiology and cancer therapy, as dysregulation of SUMOylation is associated with diverse cancers.

## MATERIALS AND METHODS

### Materials and cell culture

The A549 and LETPα-2 lung adenocarcinoma cells (LAC) cell lines were from the American Type Culture Collection (ATCC, Manassas, VA). A549 and LETPα-2 cells were cultured in Dulbecco's modified Eagle's medium (DMEM) supplemented with 10% heat-inactivated fetal bovine serum (FBS), 100 U/ml penicillin and 100 μg/ml streptomycin at 37°C under an atmosphere containing 5% CO_2_.

Lentivirus-mediated RNF4 vector (Lv-RNF4), Lv-RNF4-siRNA, negative control (NC) vector and virion-packaging elements were purchased from Genechem (Shanghai, China). The primers of RNF4 and NDRG2 were synthesized by TaKaRa (Dalian, China). The anti-RNF4, anti-NDRG2, anti-β-actin and anti-GAPDH antibodies were purchased from Cell Signaling Technologies (Boston, MA, USA).

### Reagents

DMEM and FBS were from Thermo Fisher Scientific Inc. (Waltham, MA, USA). TRIzol reagent and Lipofectamine 2000 were from Invitrogen (Carlsbad, CA, USA). M-MLV Reverse Transcriptase was from Promega (Madison, WI, USA). SYBR Green Master Mixture was from Takara (Otsu, Japan). ECL-PLUS Kit was from GE Healthcare (Piscataway, NJ, USA).

### Western blot assay

Lung adenocarcinoma cells were harvested and extracted in lysis buffer (Tris-HCl, SDS, Mercaptoethanol, Glycerol). Cell extracts were then boiled for 5 min in loading buffer, and equal aliquots of cell extract were separated on SDS-PAGE gels. The separated protein bands were then transferred into polyvinylidene fluoride membranes. After blocking the membranes in 5% skim milk, they were incubated overnight at 4°C with primary anti-RNF4, anti-NDRG2, anti-β-actin and anti-GAPDH antibodies diluted according to the manufacturer's instructions. Horseradish peroxidase-linked secondary antibodies were then added at a dilution ratio of 1:1000 and incubated fro 2 h at room temperature. The membranes were washed with PBS for three times and the immunoreactive bands were visualized using an ECL-PLUS Kit according to the kit's instruction. Relative levels of the different proteins were normalized to that of GAPDH or β-actin. Three separate experiments were performed for each clone.

### Colony formation assay

Two-layer soft agar (Sigma, UAS) colonogenic assays were conducted in a 6-well plate. A mixture of 0.5% Agar, DMEM, and 10% FBS was used for the base agar, and a mixture of 0.35% Agar, DMEM, and 10% FBS was used for the top agar. Cells stably expressing the Vector-expressing, NDRG2-WT, NDRG2-K245R and NDRG2-K333R were seeded into six-well plates in triplicate at a concentration of 5 × 10^3^ cells/well. Photomicrographs of the cells growing in plate and of the colonies developed in soft agar were taken 2 weeks after seeding. Colonies containing more than 100 cells were counted under a microscope after 2 weeks.

### Nickel pull-down assay

After transfecting A549 or LETPα-2 cells, one-tenth of the transfectants were lysed in 2 × Laemmli sample buffer (input), while the rest was lysed in buffer containing 6 M guanidine-HCl, 100 mM Na_2_HPO_4_/NaH_2_PO_4_ (pH 8), 10 mM Tris HCl (pH 8), and 10 mM imidazole. The lysates were then processed as described previously [33].

### Statistical analysis

SPSS 17.0 was used for statistical analysis. The Kruskal-Wallis *H* test and chi-squared test were used to analyze the expression rates in all groups. One-way ANOVA was used to analyze differences among groups. The LSD method of multiple comparisons was used when the ANOVA showed significant effects. Values of *P* < 0.05 were considered significant.

## References

[1] Jemal A, Bray F, Center MM, Ferlay J, Ward E, Forman D (2011). Global cancer statistics. CA-Cancer J Clin.

[2] Viktorsson K, Lewensohn R, Zhivotovsky B (2005). Apoptotic pathways and therapy resistance in human malignancies. Adv Cancer Res.

[3] Melotte V, Qu X, Ongenaert M, van Criekinge W, de Bruine AP, Baldwin HS, van Engeland M (2010). The N-myc downstream regulated gene (NDRG) family: diverse functions, multiple applications. FASEB J.

[4] Lorentzen A, Lewinsky RH, Bornholdt J, Vogel LK, Mitchelmore C (2011). Expression profile of the N-myc Downstream Regulated Gene 2 (NDRG2) in human cancers with focus on breast cancer. BMC cancer.

[5] Dandoy-Dron F, Monthioux E, Jami J, Bucchini D (1991). Regulatory regions of rat insulin I gene necessary for expression in transgenic mice. Nucleic Acids Res.

[6] Zhou B, Tang Z, Deng Y, Hou S, Liu N, Lin W, Liu X, Yao L (2014). Tumor suppressor candidate gene, NDRG2 is frequently inactivated in human glioblastoma multiforme. Mol Med Rep.

[7] Skiriute D, Tamasauskas S, Asmoniene V, Saferis V, Skauminas K, Deltuva V, Tamasauskas A (2011). Tumor grade-related NDRG2 gene expression in primary and recurrent intracranial meningiomas. J Neuro-Oncol.

[8] Lee DC, Kang YK, Kim WH, Jang YJ, Kim DJ, Park IY, Sohn BH, Sohn HA, Lee HG, Lim JS, Kim JW, Song EY, Kim DM (2008). Functional and clinical evidence for NDRG2 as a candidate suppressor of liver cancer metastasis. Cancer Res.

[9] Li R, Yu C, Jiang F, Gao L, Li J, Wang Y, Beckwith N, Yao L, Zhang J, Wu G (2013). Overexpression of N-Myc downstream-regulated gene 2 (NDRG2) regulates the proliferation and invasion of bladder cancer cells *in vitro* and *in vivo*. PloS one.

[10] Cao W, Zhang JL, Feng DY, Liu XW, Li Y, Wang LF, Yao LB, Zhang H, Zhang J (2014). The effect of adenovirus-conjugated NDRG2 on p53-mediated apoptosis of hepatocarcinoma cells through attenuation of nucleotide excision repair capacity. Biomaterials.

[11] Renner F, Moreno R, Schmitz ML (2010). SUMOylation-dependent localization of IKKepsilon in PML nuclear bodies is essential for protection against DNA-damage-triggered cell death. Mol Cell.

[12] McCool K, Miyamoto S (2009). A PAR-SUMOnious mechanism of NEMO activation. Molecular cell.

[13] Bergink S, Jentsch S (2009). Principles of ubiquitin and SUMO modifications in DNA repair. Nature.

[14] Huang TT, Wuerzberger-Davis SM, Wu ZH, Miyamoto S (2003). Sequential modification of NEMO/IKKgamma by SUMO-1 and ubiquitin mediates NF-kappaB activation by genotoxic stress. Cell.

[15] Palancade B, Doye V (2008). Sumoylating and desumoylating enzymes at nuclear pores: underpinning their unexpected duties?. Trends Cell Biol.

[16] Mokhonova EI, Avliyakulov NK, Kramerova I, Kudryashova E, Haykinson MJ, Spencer MJ (2015). The E3 ubiquitin ligase TRIM32 regulates myoblast proliferation by controlling turnover of NDRG2. Hum Mol Genet.

[17] Geiss-Friedlander R, Melchior F (2007). Concepts in sumoylation: a decade on. Nat Rev Mol Cell Bio.

[18] Hay RT (2005). SUMO: a history of modification. Mol Cell.

[19] Kim DH, Xiao Z, Kwon S, Sun X, Ryerson D, Tkac D, Ma P, Wu SY, Chiang CM, Zhou E, Xu HE, Palvimo JJ, Chen LF (2015). A dysregulated acetyl/SUMO switch of FXR promotes hepatic inflammation in obesity. EMBO J.

[20] Rajan S, Plant LD, Rabin ML, Butler MH, Goldstein SA (2005). Sumoylation silences the plasma membrane leak K+ channel K2P1. Cell.

[21] Hu XL, Liu XP, Deng YC, Lin SX, Wu L, Zhang J, Wang LF, Wang XB, Li X, Shen L, Zhang YQ, Yao LB (2006). Expression analysis of the NDRG2 gene in mouse embryonic and adult tissues. Cell Tissue Res.

[22] Lee DG, Lee SH, Kim JS, Park J, Cho YL, Kim KS, Jo DY, Song IC, Kim N, Yun HJ, Park YJ, Lee SJ, Lee HG (2015). Loss of NDRG2 promotes epithelial-mesenchymal transition of gallbladder carcinoma cells through MMP-19-mediated Slug expression. J Hepatol.

[23] Nakahata S, Ichikawa T, Maneesaay P, Saito Y, Nagai K, Tamura T, Manachai N, Yamakawa N, Hamasaki M, Kitabayashi I, Arai Y, Kanai Y, Taki T (2014). Loss of NDRG2 expression activates PI3K-AKT signalling via PTEN phosphorylation in ATLL and other cancers. Nat Commun.

[24] Dohmen RJ (2004). SUMO protein modification. Biochimica et biophysica acta.

[25] Praefcke GJ, Hofmann K, Dohmen RJ (2012). SUMO playing tag with ubiquitin. Trends Biochem Sci.

[26] Garza R, Pillus L (2013). STUbLs in chromatin and genome stability. Biopolymers.

[27] Jackson SP, Durocher D (2013). Regulation of DNA damage responses by ubiquitin and SUMO. Mol Cell.

[28] Yin Y, Seifert A, Chua JS, Maure JF, Golebiowski F, Hay RT (2012). SUMO-targeted ubiquitin E3 ligase RNF4 is required for the response of human cells to DNA damage. Gene Dev.

[29] Galanty Y, Belotserkovskaya R, Coates J, Jackson SP (2012). RNF4, a SUMO-targeted ubiquitin E3 ligase, promotes DNA double-strand break repair. Gene Dev.

[30] Vyas R, Kumar R, Clermont F, Helfricht A, Kalev P, Sotiropoulou P, Hendriks IA, Radaelli E, Hochepied T, Blanpain C, Sablina A, van Attikum H, Olsen JV (2013). RNF4 is required for DNA double-strand break repair *in vivo*. Cell Death Dis.

[31] Luo K, Zhang H, Wang L, Yuan J, Lou Z (2012). Sumoylation of MDC1 is important for proper DNA damage response. EMBO J.

[32] Park J, Kim K, Lee EJ, Seo YJ, Lim SN, Park K, Rho SB, Lee SH, Lee JH (2007). Elevated level of SUMOylated IRF-1 in tumor cells interferes with IRF-1-mediated apoptosis. Pro Natl Acad Sci USA.

[33] Rodriguez MS, Desterro JM, Lain S, Midgley CA, Lane DP, Hay RT (1999). SUMO-1 modification activates the transcriptional response of p53. EMBO J.

